# Pancreatic cancer arising in the remnant pancreas is not always a relapse of the preceding primary

**DOI:** 10.1038/s41379-018-0183-7

**Published:** 2018-11-22

**Authors:** Claudio Luchini, Antonio Pea, Jun Yu, Jin He, Roberto Salvia, Giulio Riva, Matthew J. Weiss, Claudio Bassi, John L. Cameron, Ralph H. Hruban, Michael Goggins, Christopher L. Wolfgang, Aldo Scarpa, Laura D. Wood, Rita T. Lawlor

**Affiliations:** 1 Department of Diagnostics and Public Health, Section of Pathology, University of Verona, Verona, Italy; 2 Department of Pathology, Sol Goldman Pancreatic Cancer Research Center, The Johns Hopkins University School of Medicine, Baltimore, MD USA; 3 Department of Surgery, University and Hospital Trust of Verona, Verona, Italy; 4 Department of Surgery, Sol Goldman Pancreatic Cancer Research Center, The Johns Hopkins University School of Medicine, Baltimore, MD USA; 5 Department of Oncology, Sol Goldman Pancreatic Cancer Research Center, The Johns Hopkins University School of Medicine, Baltimore, MD USA; 6 Department of Medicine, Sol Goldman Pancreatic Cancer Research Center, The Johns Hopkins University School of Medicine, Baltimore, MD USA; 7 ARC-Net Research Center, University of Verona, Verona, Italy

**Keywords:** Pancreatic cancer, Molecular biology

## Abstract

This study aimed to understand the biology of pancreatic ductal adenocarcinoma that arises in the remnant pancreas after surgical resection of a primary pancreatic ductal adenocarcinoma, using integrated histological and molecular analysis. Patients who underwent a completion pancreatectomy for local recurrence following resection of a primary pancreatic ductal adenocarcinoma were studied with histological analysis and next-generation sequencing of the primary and the recurrent cancer. Of six patients that met the inclusion criteria, three cases were classified as “true” recurrences, i.e., the primary and the cancer in the remnant pancreas shared both morphological features and molecular alterations. Two cases were identified as having independent cancers that exhibited different histological and molecular profiles. In the remaining case, the relationship could not be determined. Pancreatic ductal adenocarcinoma that arises in the remnant pancreas can be either a second primary or a “true” relapse of the preceding primary. The differentiation of second primaries from local recurrences may have important implications for patient management.

## Introduction

Pancreatic ductal adenocarcinoma represents the seventh leading cause of death for cancer in the world in the last years, causing over 300,000 deaths per year [1]. Surgical resection is the treatment of choice for localized tumor, whereas only a minority of patients presents with resectable disease at diagnosis [2]. Almost all patients undergoing surgery eventually develop local or systemic recurrence [2, 3]. Recently, different patterns and timing of recurrence have been described, highlighting the wide spectrum of biological behavior of pancreatic ductal adenocarcinoma [3]. While the majority of pancreatic ductal adenocarcinoma develops metastatic disease at a distant site, in a recent series, 24% of patients had their first site of recurrences in the remnant pancreas [3]. When local recurrence presents with a new mass in the remnant pancreas it is often difficult to distinguish intrapancreatic progression of the resected primary neoplasm from a new and independent cancer. Genetic characterization provides the opportunity to compare morphologically similar cancers in order to assess their clonal relationship. Here we studied a selected cohort of six patients undergoing pancreatic resection for pancreatic ductal adenocarcinoma, who developed a metachronous lesion in the remnant pancreas. The histopathological and molecular characteristics of the primary and metachronous lesions were compared to assess whether metachronous pancreatic ductal adenocarcinoma was an intrapancreatic metastasis or a second independent neoplasm.

## Materials and methods

### Patient selection

A retrospective review of a prospectively collected pancreatic resection database and the surgical pathology database of the Verona University Hospital Trust and Johns Hopkins Hospital was performed to identify patients with initially resected pancreatic ductal adenocarcinoma who underwent an additional resection of the remnant pancreas for a metachronous pancreatic ductal adenocarcinoma occurring at least after 12 months from the primary resection. All resections for pancreatic cystic neoplasms were excluded from the study. Verona ethics committee and the Institutional Review Board of Johns Hopkins Hospital approved the present study.

### Histopathological analysis

Following histopathological revision, pancreatic ductal adenocarcinomas were classified comparing the morphology of the primary and recurrent tumors, including grade and variant tumor types, as suggested by a recent morphological classification [4]. Recognizing as important the issue of tumor heterogeneity, we have also performed an estimate of the percentage of tumor sampling following the methods described by a previous study on this topic [5].

### Next-generation sequencing

DNA was obtained from tumor and matched normal formalin-fixed paraffin-embedded tissue blocks, after enrichment for neoplastic cellularity to at least 80% using manual microdissection of 10 consecutive 4-μm formalin-fixed paraffin-embedded sections. The molecular analysis was conducted as already described [6]. Briefly, DNA was extracted using QIAamp DNA Micro Kit (Qiagen). Genomic DNA was quantified by Quantifiler Human DNA Quantification kit (Applied Biosystems). Twenty nanograms of genomic DNA was amplified using Ampliseq reagents for the library preparation, loaded and sequenced onto 318v2 chips using an Ion Torrent Personal Genome Machine (PGM, Life Technologies) following the manufacturer’s protocols. Post-sequencing data analyses were performed using NextGENe software (v2.4, SoftGenetics, Chicago, IL). Alignments and putative mutations were visually checked using the Integrative Genomics Viewer (IGV, v2.3, Broad Institute) and the NextGENeViewer (v2.4, SoftGenetics, Chicago, IL). The Ion AmpliSeq Custom Panel and Ion AmpliSeq Designer (Pipeline version 4.2, Life technologies) were employed to perform multiplex PCR and sequencing of 11 genes (142 amplicons in 2 primer pools), known to be targeted in pancreatic ductal adenocarcinoma (*KRAS, TP53, SMAD4, CDKN2A, GNAS, RNF43, TGFBR2, ARID1A, BRAF, MAP2K4 and PIK3CA*) (Supplementary Table 1) [7].

### Classification of the recurrence

Histopathological and molecular analyses were integrated in order to assess the nature of the metachronous lesion in the remnant pancreas. Particularly, when the histological and molecular patterns were concordant between the primary and the relapse lesion, this was defined as a “true” intrapancreatic recurrence of the primary pancreatic ductal adenocarcinoma. Conversely, the secondary lesion was defined as “independent” when it presented different morphological and molecular profiles from the primary lesion. The relationship was considered as “undetermined”, when its assessment was not possible according to the aforementioned criteria.

## Results

### Patients

Upon the analysis of clinical records at Verona University Hospital and Johns Hopkins Hospital, six patients who underwent a completion pancreatectomy for pancreatic ductal adenocarcinoma following resection of a primary pancreatic ductal adenocarcinoma, with complete follow-up information and available formalin-fixed paraffin-embedded tissues, were identified. The main characteristics of these patients are summarized in Table 1. All patients presented a second pancreatic ductal adenocarcinoma in the pancreatic remnant, which was localized to the pancreas and suitable for surgical resection. The mean time of development of the second lesion was 37 months. Four of the six patients were females and two were males. The mean age of the patients was 63.8 years, and no patients declared familial history of pancreatic ductal adenocarcinoma. Two patients were classified as R1 at the first operation in spite of negativity of intraoperative frozen sections (pancreatic neck and uncinate process) as at the final histopathological evaluation demonstrated the involvement of the resection margin close to the vascular groove (where the portal vein-superior mesenteric vein passes behind the pancreas).

**Table 1:** Clinical, pathological and genetic characteristics of cases with primary PDAC that underwent a completion pancreatectomy for development of a second PDAC in the remnant pancreas

Patient, age, gender	1st PDAC						*R*	Positive margin	Months after 1st op	2nd PDAC						Classification of recurrence
	Operation	G	T	N	Histology	Mutations				Location	G	T	N	Histology	Mutations	
1) 72, f	Whipple	2	2	1	Conventional	*KRAS*: p.G12D *TP53*: p.Q104X	1	Mesenteric vein	38	Peri - anastomotic	2	2	1	Conventional	*KRAS*: p.G12D *TP53*: p.Q104X	True Recurrence
2) 74, f	Whipple	2	2	1	Conventional	*KRAS*: p.G12V	0	/	50	2, 3 cm from anastomosis	3	2	0	UND with osteoclast cells	*KRAS*: p.G12D *TP53*: p.R290Pfs	Independent
3) 67, f	DP	3	3	1	Conventional	*KRAS*: p.G12R	0	/	36	Head	3	2	0	Conventional	*KRAS*: p.G12R	True Recurrence
4) 63, m	Whipple	2	3	1	Cribriform	*KRAS*: p.G12V	0	/	48	Distal tail	2	2	1	Conventional	*KRAS*: p.G12D	Independent
5) 54, m	Whipple	2	3	1	Conventional	*KRAS*: p.G12D	0	/	36	1, 5 cm from anastomosis	2	2	1	Micropapillary	*KRAS*: p.G12D	Undetermined
6) 53, f	Whipple	3	2	1	Conventional	*KRAS*: p.G12R	1	Mesenteric vein	16	Peri - anastomotic	2	2	1	Conventional	*KRAS*: p.G12R	True Recurrence
										Distal tail	3	1c	1	Conventional	*KRAS*: p.G12R	

*DP* distal pancreatectomy, *G* tumor grading, *T* pT stage, *N* pN stage, *UND* undifferentiated

All patients underwent adjuvant standard chemotherapy following the first surgical operation; only one patient (case #6) also received radiotherapy.

### Histopathological analysis

Five primary cancers (cases #1, #2, #3, #5, and #6) had conventional pancreatic ductal adenocarcinoma histology; among these, in three cases (#1, #3, and #6) the matched recurrent tumors displayed the same conventional ductal adenocarcinoma morphology. In the remaining cases, the subsequent cancer resection had an undifferentiated carcinoma with osteoclast-like giant cells associated with a classical ductal adenocarcinoma component (case #2), and a pancreatic ductal adenocarcinoma with micropapillary features in the other one (case #5) (Table 1).

The remaining primary pancreatic ductal adenocarcinoma (case #4) had cribriform features, and its matched recurrent tumor had a conventional pancreatic ductal adenocarcinoma, poorly differentiated.

Regarding the extent of tumor sampling, all tumors comprised in our series were either entirely included (primary tumors of cases #3 and #4, recurrent tumors of cases #1, #3, #4, #5, and one of the two recurrences of case #6), or extensively sampled (primary tumors: 80% case #1; 90% case #2; 90% case #5; 80% case #6; 90% case #2, 80% the remaining recurrence of case #6).

### Molecular analysis

In four cases (#1, #3, #5, and #6), the same *KRAS* mutations were present in both the primary and the matched recurrent cancer. Case #1 displayed the same *KRAS* and *TP53* mutations in both cancers.

Two cases (#2 and #4) had different *KRAS* mutations in the primary and recurrent cancer; one of these (case #2) displayed an additional *TP53* mutation in the recurrence that was not present in the primary. All other targeted genes were not mutated.

### Classification of the recurrence

Integrating morphological and molecular data, three cases (#1, #3 and #6) showed a perfect correspondence between the primary tumor and its matched recurrence. In these cases, we classified the second cancer as a “true” recurrence. The mean time to develop the second lesion was of 30 months. Two of these cases (#1 and #6) were those classified as R1 at the time of the first surgical resection.

Two other cases (case #2 and #4) showed different morphological and molecular profiles. Here the metachronous tumor has been classified as an independent pancreatic ductal adenocarcinoma. In these cases, the mean time to develop the second lesion was of 49 months.

The remaining case (#5) displayed the same *KRAS* mutation between the primary and the recurrence, but a different morphology, being the primary a conventional pancreatic ductal adenocarcinoma and the relapse a pancreatic ductal adenocarcinoma with micropapillary features. In this case, the recurrence was considered as an undetermined lesion, and occurred 36 months after the first operation.

## Discussion

In this study, we have dissected the relationship between metachronous pancreatic ductal adenocarcinoma lesions arising in the same pancreas. These cancers were temporally separated and separately resected. Using an integrated histopathological and molecular approach, we describe two distinct potential processes for metachronous pancreatic ductal adenocarcinoma following a primary pancreatic resection, some are true recurrence of the initial primary in the remnant pancreas, while others are true second primaries.

### “True” relapse in R1 resection

The first mechanism depends on surgical positive margins (cases #1 and #6): the residual neoplasm grows from residual disease left behind at the first operation. The correspondence of morphological (conventional pancreatic ductal adenocarcinoma) and molecular features between the primary tumors and the recurrences indicates that the recurrent pancreatic ductal adenocarcinoma represents a “true recurrence”, and the presence of positive resection margins strongly supports this classification, since R1 pancreatic resection has been already described as a major risk factor for pancreatic ductal adenocarcinoma local recurrence [8, 9]. Case #1 shows the same *KRAS* and *TP53* mutations between the primary tumor and the recurrence, and case #6 has the same *KRAS* G12R mutation in the primary and in both metachronous pancreatic ductal adenocarcinomas. We acknowledge that sharing the same *KRAS* mutation alone does not guarantee genetic relatedness, but the G12R mutation is relatively uncommon (only ~10–15% of pancreatic ductal adenocarcinomas). In addition, the morphological patterns in the primary and recurrence were the same in this case, and the same *KRAS* G12R mutation is shared by the primary tumor and two different recurrences [7, 10].

Recurrence of the primary in the remnant pancreas, as demonstrated by case #3, can also occur by intraparenchymal metastases: it represents the second potential mechanism of cancer development in the pancreatic remnant. Although the surgical margins were negative in the first operation in this case and the metachronous lesion was physically separated from the pancreatic anastomosis, the primary and the recurrent pancreatic ductal adenocarcinoma are probably related, sharing the same morphological features and the same G12R *KRAS* mutation. Although sharing the same conventional morphology and the same *KRAS* mutation does not definitively demonstrate relatedness, the primary and the recurrent lesions are likely related, due to the morphological similarities as well as sharing the less common G12R mutation in *KRAS*. This case may demonstrate intrapancreatic (intraductal or intraparenchymal) metastasis.

### New independent pancreatic ductal adenocarcinoma

The third mechanism is suggested by the metachronous lesions in cases #2 and #4. In fact, the metachronous lesions in these cases are seemingly independent from the corresponding primary tumor, due to both the different genetic alterations involving driver genes and the different histological features. Notably, the second lesion of case #2 is an undifferentiated carcinoma with osteoclast-like giant cells, a tumor type that has been recently demonstrated by whole-exome sequencing to be a real variant of conventional ductal adenocarcinoma [11], and for this reason it has been included in the present series.

In a “high-risk” pancreas, i.e., a pancreas in which a pancreatic ductal adenocarcinoma has been already developed, a second pancreatic ductal adenocarcinoma may develop in a different location in the pancreatic remnant. The second lesion is genetically independent from the primary pancreatic ductal adenocarcinoma, and represents the result of a second complete and independent pancreatic ductal adenocarcinoma tumorigenesis in the same pancreas, as we show in cases #2 and # 4. The mean time of development of a new pancreatic ductal adenocarcinoma (49 months) was longer than that observed for “true” recurrences (30 months). It is worth of note that there are mutliple reasons why mutliple independent primary pancreatic ductal adenocarcinomas are not frequently observed clinically. Independent primaries are unlikely to both reach the point of clinical attention at the same time, and thus are unlikely to be synchronous (i.e., identified in the same surgical resection specimen). Furthermore, metachronous lesions (which might represent independent primaries as we show in this case-series) are unlikely to be resected, mainly due to the local and/or distant diffusion of the disease, thus such relatedness is under-studied in the literature. Thus, although rare, the situation observed in our cases #2 and #4 is paradigmatic for the possible independence between primary and metachronous pancreatic ductal adenocarcinomas.

The recurrence of case #5 has been considered as “undetermined”, but it is likely to be an independent lesion due to the different morphology of the two lesions (conventional the primary and with micropapillary features the recurrence). The primary and the recurrent tumors share the same G12D *KRAS* mutation (very common in pancreatic ductal adenocarcinoma), but their different morphology suggests discordant mutations in genes that were not considered in our panel of 11 genes.

Similar findings have been reported for recurrent intraductal papillary mucinous neoplasms [12, 13]. Pea et al., furthermore, described that some recurrent intraductal papillary mucinous neoplasms are caused by intraductal spread of a single neoplasm, while other recurrent intraductal papillary mucinous neoplasms are likely independent from the primary resected intraductal papillary mucinous neoplasms [6]. Along this line, Felsenstein et al., using a specific panel of cancer driver genes, demonstrated that even co-occurring intraductal papillary mucinous neoplasm and pancreatic ductal adenocarcinoma in the same pancreas are genetically distinct in about 20% of cases [14]. Other studies show that more precursor lesions are present in the pancreata of patients with familial pancreatic cancer than in pancreas of patients without family history, and that germline mutations in pancreatic ductal adenocarcinoma susceptibility genes are commonly identified in patients with pancreatic ductal adenocarcinoma lacking a family history for this tumor [15, 16].

Taken together, these findings call for new considerations about the use of molecular analysis to determine the relatedness of recurrent precancers and cancers after pancreatic resection, as some represent regrowth of the previously resected neoplasm, while others the development of a second primary lesion [15]. A limitation of our study is that this cohort comprises few cases, since most local recurrences are identified when they are too advanced for surgery.

**Fig. 1:**
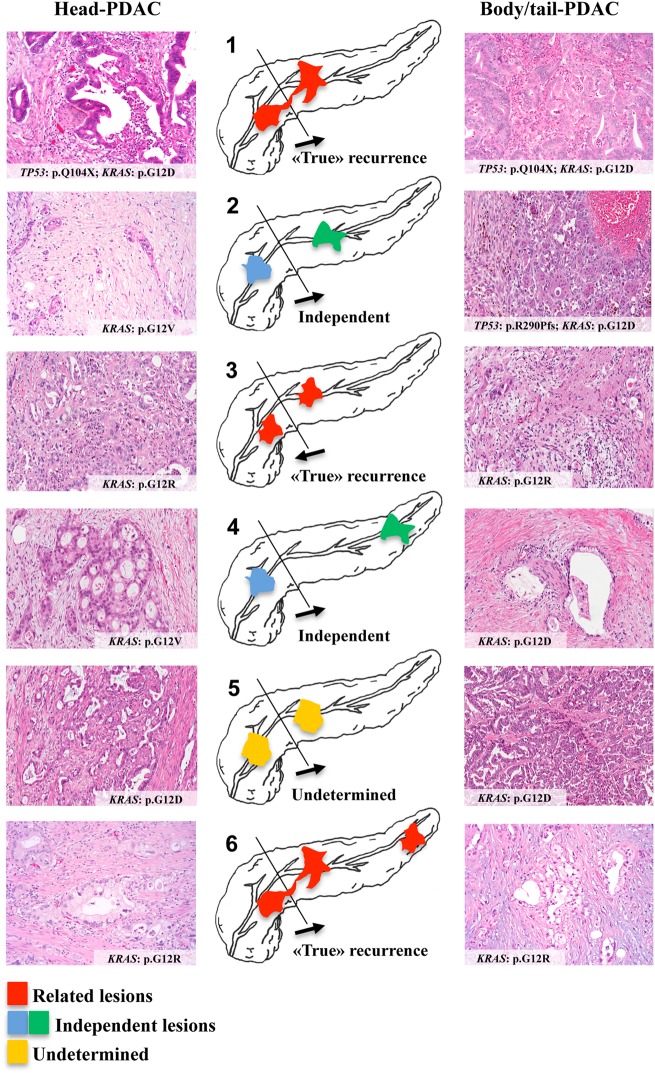
Graphical summary. The relationship between the six primary and recurrent pancreatic ductal adenocarcinoma (PDAC), with their histological and molecular features, is here shown. The cases have been numbered according to the text. The direction of the arrows indicates whether the first pancreatic ductal adenocarcinoma affected the head and then the tail (cases #1, #2, #4, #5, and #6) or vice versa (case #3). The lesions have been colored in red in case of “true” recurrence, in blue and green in case of independent lesions, and in yellow in case of  undetermined recurrence

A relevant issue regarding our interpretation of the nature of the lesion found in remnant pancreas is represented by tumor heterogeneity, which can infer a possible bias in determining its relationship with the primary tumor. To address this, we have documented an extensive sampling of the tumors of our series, limiting the potential inaccuracy in assessing tumor morphology. Furthermore, recent seminal papers have clarified that there is a limited intra-tumor heterogeneity with respect to driver gene mutations in pancreatic ductal adenocarcinoma [17–19]. Based on these observations, our targeted analysis investigating the most important driver genes in pancreatic cancer appears reliable in representing the actual molecular profile of the analyzed lesions (Fig. 1).

It is also of interest to consider the modifications that adjuvant treatments can induce on tumor morphology. Given that chemo-radiation therapy may induce changes in morphology [20], we used an integrated approach with both morphology and molecular analysis. As previously discussed regarding the issue of tumor heterogeneity, driver gene mutations are homogeneous within a pancreatic ductal adenocarcinoma [17], thus even if a second lesion derived from a minor molecular subclone it will still have the same *KRAS* mutation as the primary tumor sample. Therefore, the classification of lesions as independent appears reliable and not due to chemotherapy effect.

We acknowledge as a limitation of our study the use of R1 status in favor of a true recurrence, which may not be always unfailing. Indeed, also depending on the modalities of surgical resection, a significant proportion of pancreatic ductal adenocarcinomas shows neoplastic cells in the tissues covering the gland [21]. This phenomenon can partly explain the early dissemination after the resection, not rarely seen regardless the status of margins. Although this observation appears of importance, in our series the presence of a positive resection margin emerged as a reliable risk factor for the local recurrence of pancreatic ductal adenocarcinoma.

Our results provide new insights into the biology of pancreatic ductal adenocarcinoma recurrence. The determination that recurrence of pancreatic ductal adenocarcinoma in the remnant can represent a second primary may have important implications for the management of patients with recurrent pancreatic ductal adenocarcinoma. Further studies are needed to determine the clinical utility of distinguishing intraparenchymal spread from second primaries, and to better characterize pancreas at high risk to develop neoplasms along the entire gland. Patients at increased risk would benefit from the resection of the entire gland and/or from personalized surveillance approaches.

## Electronic supplementary material

Supplementary Table 1
