# Supplementary material for: Pancreatic cancer arising in the remnant pancreas is not always a relapse of the preceding primary
Source: Mod Pathol. 2018 Nov 22;32(5):659–65. doi: 10.1038/s41379-018-0183-7 (PMC6760648; doi:10.1038/s41379-018-0183-7)
Supplement: Supplementary file 1 — Supplementary Table 1 [file 41379_2018_183_MOESM1_ESM.docx]

| **Supplementary Table 1.** Targeted regions of the analyzed 11 genes explored by the AmpliSeq custom panel. | | |  |
| --- | --- | --- | --- |
|  | | |  |
| **Chromosome** | **Chr Start** | **Chr_End** | **Gene** |
| chr1 | 27022973 | 27023197 | *ARID1A* |
| chr1 | 27023202 | 27023404 | *ARID1A* |
| chr1 | 27023412 | 27023521 | *ARID1A* |
| chr1 | 27023535 | 27023683 | *ARID1A* |
| chr1 | 27023793 | 27024029 | *ARID1A* |
| chr1 | 27056089 | 27056214 | *ARID1A* |
| chr1 | 27056191 | 27056416 | *ARID1A* |
| chr1 | 27057577 | 27057805 | *ARID1A* |
| chr1 | 27057793 | 27057961 | *ARID1A* |
| chr1 | 27057972 | 27058195 | *ARID1A* |
| chr1 | 27059113 | 27059335 | *ARID1A* |
| chr1 | 27087239 | 27087434 | *ARID1A* |
| chr1 | 27087426 | 27087640 | *ARID1A* |
| chr1 | 27087806 | 27088033 | *ARID1A* |
| chr1 | 27088611 | 27088835 | *ARID1A* |
| chr1 | 27089438 | 27089662 | *ARID1A* |
| chr1 | 27089665 | 27089875 | *ARID1A* |
| chr1 | 27092654 | 27092779 | *ARID1A* |
| chr1 | 27092768 | 27092862 | *ARID1A* |
| chr1 | 27092933 | 27093050 | *ARID1A* |
| chr1 | 27093012 | 27093135 | *ARID1A* |
| chr1 | 27094247 | 27094476 | *ARID1A* |
| chr1 | 27097538 | 27097761 | *ARID1A* |
| chr1 | 27097725 | 27097950 | *ARID1A* |
| chr1 | 27098942 | 27099168 | *ARID1A* |
| chr1 | 27099254 | 27099363 | *ARID1A* |
| chr1 | 27099364 | 27099483 | *ARID1A* |
| chr1 | 27099800 | 27100027 | *ARID1A* |
| chr1 | 27100040 | 27100153 | *ARID1A* |
| chr1 | 27100142 | 27100233 | *ARID1A* |
| chr1 | 27100292 | 27100418 | *ARID1A* |
| chr1 | 27100776 | 27101006 | *ARID1A* |
| chr1 | 27101007 | 27101170 | *ARID1A* |
| chr1 | 27101092 | 27101283 | *ARID1A* |
| chr1 | 27101321 | 27101523 | *ARID1A* |
| chr1 | 27101559 | 27101788 | *ARID1A* |
| chr1 | 27102062 | 27102190 | *ARID1A* |
| chr1 | 27105461 | 27105677 | *ARID1A* |
| chr1 | 27105654 | 27105848 | *ARID1A* |
| chr1 | 27105884 | 27106097 | *ARID1A* |
| chr1 | 27106113 | 27106241 | *ARID1A* |
| chr1 | 27106255 | 27106479 | *ARID1A* |
| chr1 | 27106483 | 27106625 | *ARID1A* |
| chr1 | 27106618 | 27106848 | *ARID1A* |
| chr1 | 27106836 | 27107063 | *ARID1A* |
| chr1 | 27107082 | 27107307 | *ARID1A* |
| chr3 | 30648361 | 30648459 | *TGFBR2* |
| chr3 | 30664683 | 30664805 | *TGFBR2* |
| chr3 | 30686252 | 30686455 | *TGFBR2* |
| chr3 | 30691752 | 30691962 | *TGFBR2* |
| chr3 | 30713154 | 30713377 | *TGFBR2* |
| chr3 | 30713356 | 30713511 | *TGFBR2* |
| chr3 | 30713455 | 30713630 | *TGFBR2* |
| chr3 | 30713592 | 30713816 | *TGFBR2* |
| chr3 | 30713767 | 30713995 | *TGFBR2* |
| chr3 | 30715562 | 30715783 | *TGFBR2* |
| chr3 | 30729825 | 30730051 | *TGFBR2* |
| chr3 | 30732903 | 30733134 | *TGFBR2* |
| chr3 | 178916807 | 178916944 | *PIK3CA* |
| chr3 | 178921464 | 178921570 | *PIK3CA* |
| chr3 | 178927901 | 178927986 | *PIK3CA* |
| chr3 | 178928075 | 178928156 | *PIK3CA* |
| chr3 | 178936019 | 178936122 | *PIK3CA* |
| chr3 | 178938787 | 178938918 | *PIK3CA* |
| chr3 | 178951933 | 178952153 | *PIK3CA* |
| chr7 | 140453108 | 140453215 | *BRAF* |
| chr7 | 140481391 | 140481515 | *BRAF* |
| chr9 | 21970895 | 21971073 | *CDKN2A* |
| chr9 | 21971049 | 21971219 | *CDKN2A* |
| chr9 | 21974607 | 21974792 | *CDKN2A* |
| chr9 | 21974790 | 21974969 | *CDKN2A* |
| chr12 | 25378549 | 25378658 | *KRAS* |
| chr12 | 25380260 | 25380364 | *KRAS* |
| chr12 | 25398186 | 25398304 | *KRAS* |
| chr17 | 7572847 | 7572976 | *TP53* |
| chr17 | 7573923 | 7574035 | *TP53* |
| chr17 | 7576494 | 7576597 | *TP53* |
| chr17 | 7576578 | 7576702 | *TP53* |
| chr17 | 7576829 | 7576949 | *TP53* |
| chr17 | 7577015 | 7577151 | *TP53* |
| chr17 | 7577479 | 7577618 | *TP53* |
| chr17 | 7578180 | 7578298 | *TP53* |
| chr17 | 7578304 | 7578439 | *TP53* |
| chr17 | 7578430 | 7578560 | *TP53* |
| chr17 | 7579276 | 7579391 | *TP53* |
| chr17 | 7579389 | 7579515 | *TP53* |
| chr17 | 7579508 | 7579588 | *TP53* |
| chr17 | 7579575 | 7579684 | *TP53* |
| chr17 | 7579839 | 7579960 | *TP53* |
| chr17 | 11924202 | 11924312 | *MAP2K4* |
| chr17 | 11958120 | 11958328 | *MAP2K4* |
| chr17 | 11984665 | 11984768 | *MAP2K4* |
| chr17 | 11984767 | 11984894 | *MAP2K4* |
| chr17 | 11998829 | 11999053 | *MAP2K4* |
| chr17 | 12011075 | 12011179 | *MAP2K4* |
| chr17 | 12011144 | 12011253 | *MAP2K4* |
| chr17 | 12013608 | 12013800 | *MAP2K4* |
| chr17 | 12016508 | 12016731 | *MAP2K4* |
| chr17 | 12028525 | 12028742 | *MAP2K4* |
| chr17 | 12032431 | 12032650 | *MAP2K4* |
| chr17 | 12043044 | 12043261 | *MAP2K4* |
| chr17 | 12044425 | 12044645 | *MAP2K4* |
| chr17 | 56432208 | 56432333 | *RNF43* |
| chr17 | 56434825 | 56434956 | *RNF43* |
| chr17 | 56434984 | 56435092 | *RNF43* |
| chr17 | 56435078 | 56435209 | *RNF43* |
| chr17 | 56435208 | 56435324 | *RNF43* |
| chr17 | 56435364 | 56435595 | *RNF43* |
| chr17 | 56435507 | 56435702 | *RNF43* |
| chr17 | 56435691 | 56435865 | *RNF43* |
| chr17 | 56435879 | 56436110 | *RNF43* |
| chr17 | 56436145 | 56436218 | *RNF43* |
| chr17 | 56437464 | 56437658 | *RNF43* |
| chr17 | 56438133 | 56438366 | *RNF43* |
| chr17 | 56439846 | 56439974 | *RNF43* |
| chr17 | 56439964 | 56440058 | *RNF43* |
| chr17 | 56440603 | 56440724 | *RNF43* |
| chr17 | 56440719 | 56440832 | *RNF43* |
| chr17 | 56440875 | 56440973 | *RNF43* |
| chr17 | 56448183 | 56448410 | *RNF43* |
| chr17 | 56492607 | 56492814 | *RNF43* |
| chr17 | 56492751 | 56492979 | *RNF43* |
| chr18 | 48573387 | 48573505 | *SMAD4* |
| chr18 | 48573468 | 48573588 | *SMAD4* |
| chr18 | 48573568 | 48573675 | *SMAD4* |
| chr18 | 48575042 | 48575158 | *SMAD4* |
| chr18 | 48575135 | 48575238 | *SMAD4* |
| chr18 | 48575573 | 48575682 | *SMAD4* |
| chr18 | 48575676 | 48575749 | *SMAD4* |
| chr18 | 48581171 | 48581259 | *SMAD4* |
| chr18 | 48581248 | 48581368 | *SMAD4* |
| chr18 | 48584489 | 48584654 | *SMAD4* |
| chr18 | 48584701 | 48584831 | *SMAD4* |
| chr18 | 48586243 | 48586349 | *SMAD4* |
| chr18 | 48591735 | 48591858 | *SMAD4* |
| chr18 | 48591847 | 48591964 | *SMAD4* |
| chr18 | 48593399 | 48593519 | *SMAD4* |
| chr18 | 48603022 | 48603205 | *SMAD4* |
| chr18 | 48604602 | 48604832 | *SMAD4* |
| chr20 | 57480364 | 57480591 | *GNAS* |
| chr20 | 57484396 | 57484504 | *GNAS* |
| chr20 | 57484562 | 57484672 | *GNAS* |
